# Current and future trends of acupuncture as an adjuvant therapy in cancer: A bibliometric and visual analysis

**DOI:** 10.1097/MD.0000000000038663

**Published:** 2024-07-19

**Authors:** Rui Shang, Ting Pan, Fengyang Wang, Hui Jin, Xi Nan, Chenyu Song

**Affiliations:** a JiLin Cancer Hospital, Changchun, Jilin Province, China; b The Affiliated Hospital of Changchun University of Chinese Medicine, Changchun, Jilin Province, China; c Changchun University, Changchun, Jilin Province, China.

**Keywords:** acupuncture, bibliometric analysis, cancer, current state of research

## Abstract

**Background:**

Acupuncture, as one of the representatives of complementary therapies, is often used as an adjunctive therapy in the treatment of cancer and related complications with beneficial results.

**Methods:**

We selected the Web of Science Core Collection (WOSCC) database as the data source and searched to obtain relevant literatures on the application of acupuncture in cancer treatment. The relevant literature was analyzed using CiteSpace (version 6.1.R6) and VOSviewer (version 1.6.20).

**Results:**

A total of 592 publications were included, with an overall upward trend in the number of publications. There were 135 countries or institutions involved, with China as the most published country. There were 1888 related institutions, and Memorial Sloan Kettering Cancer Center was the institution with the most publications. A total of 3067 authors were involved, and there were obvious collaborations between authors. There were 190 related journals, and J Clin Oncol was the most cited journal. The most frequently occurring keywords were breast cancer and management, but also other related topics such as quality of life, chemotherapy, and complementary.

**Conclusion:**

Acupuncture is gaining increasing attention as an adjunctive therapy in cancer treatment programs. Currently, relevant research focuses on acupuncture to alleviate cancer-induced comorbid symptoms, such as pain and insomnia. Future research is gradually shifting toward spiritual care, Colorectal Cancer Surgery, and systematic review.

## 1. Introduction

Cancer is the leading cause of death^[1]^ and a study shows that countries with a low Human Development Index are facing a growing cancer burden.^[2]^ There is no doubt that the high incidence of cancer and the low cure rate put a huge strain on the global health system.^[3,4]^ In the data released by the World Health Organization in 2019, cancer is the first or second leading cause of death before the age of 70 in 112 out of 183 countries and the third or fourth leading cause in another 23 countries.^[5]^ In the face of this situation, more and more scholars have begun to pay attention to the mechanism of cancer and the innovation of treatment programs,^[5–7]^ and have achieved many research results. Renewal of cancer treatment programs, as well as postoperative adverse effects and complications of cancer, are all topics of concern and urgent problems.

Acupuncture is one of the therapeutic methods of traditional Chinese medicine with an ancient history, characterized by easy operation and high acceptance by patients. Currently, acupuncture is gradually becoming involved in the treatment of cancer as an adjunctive therapy., and it has good effects on improving the complications of cancer patients, such as fatigue and pain.^[8–11]^ There is a growing body of evidence showing that the use of acupuncture has positive implications for improving cancer-related symptoms and enhancing the quality of life of cancer patients.^[12,13]^ Although this state of affairs is gradually gaining attention among scholars, there has not been a systematic review to organize and summarize the research in this area in detail. Therefore, this study summarizes and combs the research in this field through bibliometric analysis methods to explore the current research hotspots and future research trends.

## 2. Materials and methods

### 2.1. Search strategy

We searched the Web of Science Core Collection (WOSCC) for literature on acupuncture for cancer. Search terms included “Acupuncture” and synonyms, “Cancer” and synonyms, etc. The publication period was limited to the year 2023. We finally retrieved 2561 articles. Table 1 demonstrates the search strategy.

**Table 1:** Publication retrieval strategy for acupuncture treatment of cancer.

Rank	Search strategy	Search results
#1	“Acupuncture” OR “Acupuncture Treatment” OR “Acupuncture Treatments” OR “Treatment, Acupuncture” OR “Therapy, Acupuncture” OR “Electroacupuncture” OR “Manual acupuncture”	29395
#2	“Cancer” OR “Cancers” OR “Tumor” OR “Tumors” OR “Neoplasm” OR “Neoplasia” OR “Neoplasias” OR “Malignant Neoplasm” OR “Malignancy” OR “Malignancies” OR “Malignant Neoplasms” OR “Neoplasm, Malignant” OR “Neoplasms, Malignant” OR “Benign Neoplasms” OR “Benign Neoplasm” OR “Neoplasms, Benign” OR “Neoplasm, Benign”	4328788
#3	#1 and #2	2561

### 2.2. Inclusion criteria

We included relevant literature published in different academic journals related to acupuncture and cancer. The language of the articles was limited to English and the type of literature was limited to articles and reviews.

### 2.3. Exclusion criteria

We will exclude consultation guides, letters, abstracts, editorials, dissertations, biographies, book reviews, conferences, news, duplicates, publications withdrawn by the authors, etc, which are not relevant to the field of study.

### 2.4. Literature screening process

**Figure 1.**
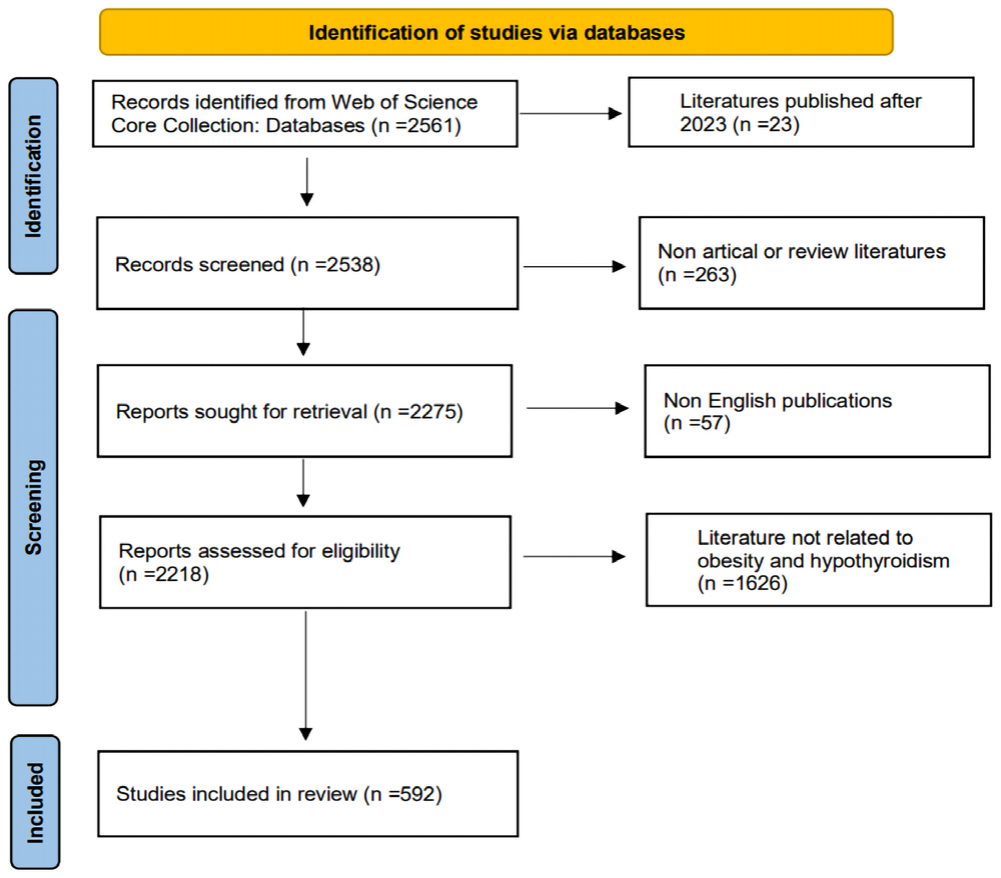
Literature selection process.

Two researchers screened the retrieved literature based on the inclusion criteria, and exclusion criteria, and finally 592 publications were included. Figure 1 illustrates the screening process.

### 2.5. Bibliometric analysis software

We used 2 software packages, CiteSpace (version 6.1.R6) and VOSviewer (version 1.6.20) for bibliometric analysis and visualization. We focused on publishing trends, countries/regions, research institutions, authors, keywords co-occurrence and clustering relationships, and forming network analysis views.

## 3. Results

### 3.1. Publication trends

**Figure 2.**
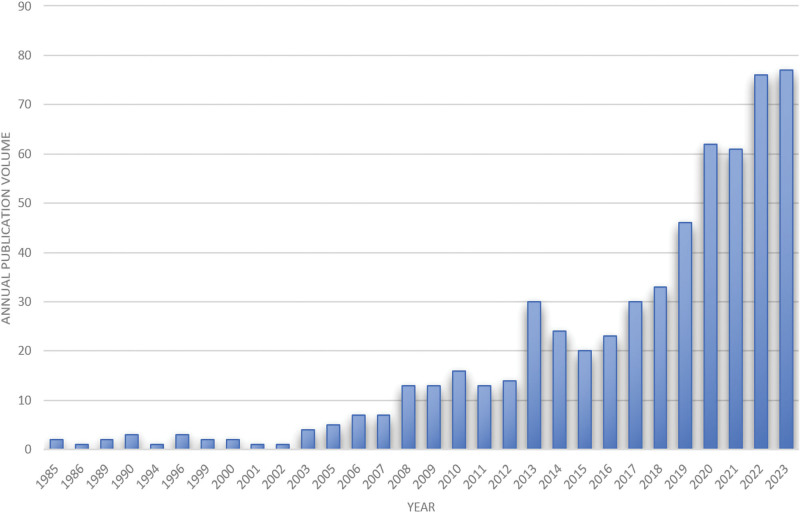
Trend of document volume.

Figure 2 shows the number of publications between 1985 and 2023, with an overall upward trend. The number of publications between 1985 and 2007 is relatively low, with an average of no more than 10 publications per year. The number of publications between 2008 and 2013 shows an increasing trend and reached 30 in 2013. The number of publications between 2013 and 2020 is no <20 per year and exceeds 60 in 2020. After 2020, the number of publications shows a clear upward trend, reaching a peak of over 80 in 2023.

### 3.2. Countries/regions analysis

**Figure 3.**
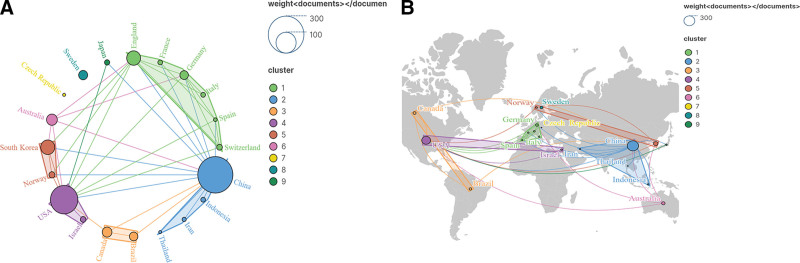
Visual analysis of countries/regions. (A) Network view analysis. (B) Countries distribution map.

A total of 135 countries/regions are involved in the 592 papers, and Table 2 shows the top 10 countries in terms of the number of papers issued. In Figure 3A, we analyzed the network view of countries/regions, and it can be seen that there is a close cooperation between these countries/regions, which has formed a remarkable cooperation cluster. Among them, China has published the largest number of related publications, reaching 249. USA has the second-largest number of publications, reaching 167. It is followed by South Korea and England with 44 and 43 publications respectively. The network view shows that these 4 countries are at the center of their respective collaborative clusters. Figure 3B illustrates the geographical distribution of these countries, and it can be seen that these countries are distributed in 5 continents around the world, and scholars from each continent have also formed stable collaborations, such as USA-Israel, Canada-Brazil, and China-Indonesia-Iran. China and the United States are also at the core of Asia and the Americas.

**Table 2:** Top 10 countries/region in terms of publication volume.

Rank	Count	Country/地区
1	249	China
2	167	USA
3	44	South Korea
4	43	England
5	27	Taiwan
6	26	Australia
7	21	Canada
8	18	Sweden
9	16	Germany
10	16	Brazil

### 3.3. Institutions analysis

**Figure 4.**
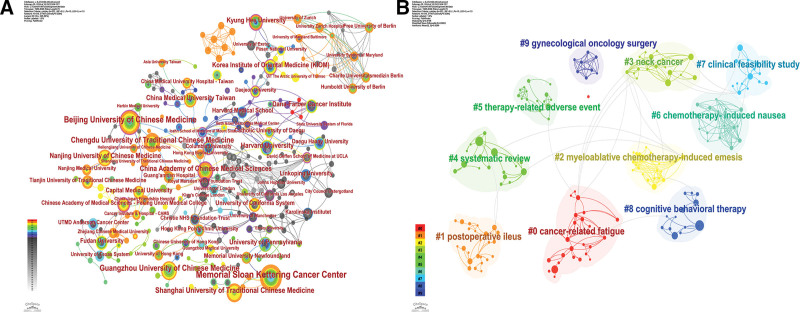
Network analysis view of publishing institutions. (A) Co-occurrence analysis view of institutions. (B) Cluster analysis view of institutions.

There are a total of 1888 institutions associated with publications, and Table 3 demonstrates the top 13 institutions in terms of the number of publications, with no <14 publications per institution. The institution with the highest number of publications was the Memorial Sloan Kettering Cancer Center, with a total of 43 publications. The institutions with the second and third largest number of publications were Beijing University of Chinese Medicine and Guangzhou University of Chinese Medicine, with 33 and 24 publications, respectively. When analyzing the institutions by centrality, the China Academy of Chinese Medical Sciences ranked first with 0.21. Korea Institute of Oriental Medicine ranked second with 0.15, followed by Beijing University of Chinese Medicine with 0.12. Figure 4A shows the co-occurrence analysis view of the institutions, with the size of the nodes representing the number of publications.

**Table 3:** Top 13 Institutions with the highest number of publications.

Rank	Count	Centrality	Institutions
1	43	0.05	Memorial Sloan Kettering Cancer Center
2	33	0.12	Beijing University of Chinese Medicine
3	24	0.01	Guangzhou University of Chinese Medicine
4	22	0.01	Chengdu University of Traditional Chinese Medicine
5	21	0.02	Shanghai University of Traditional Chinese Medicine
6	17	0.21	China Academy of Chinese Medical Sciences
7	16	0.02	Harvard University
8	16	0	Nanjing University of Chinese Medicine
9	15	0.03	China Medical University Taiwan
10	14	0.15	Korea Institute of Oriental Medicine (KIOM)
11	14	0.1	Kyung Hee University
12	14	0.05	University of Pennsylvania
13	14	0.09	Dana-Farber Cancer Institute

Figure 4B shows the results of the cluster analysis between institutions, and it can be seen that these institutions can form 10 significant clusters, which suggests that there is a relatively close collaboration between institutions. The cluster with the highest heat level is cancer-related fatigue, followed by postoperative ileus and myeloablative chemotherapy-induced emesis. this reflects the fact that the current research on this topic is mainly focused on cancer-induced complications and adverse reactions caused by surgery, chemotherapy, and other therapeutic modalities.

### 3.4. Authors analysis

#### 3.4.1. Analysis of authors’ publications and authors collaborations

**Figure 5.**
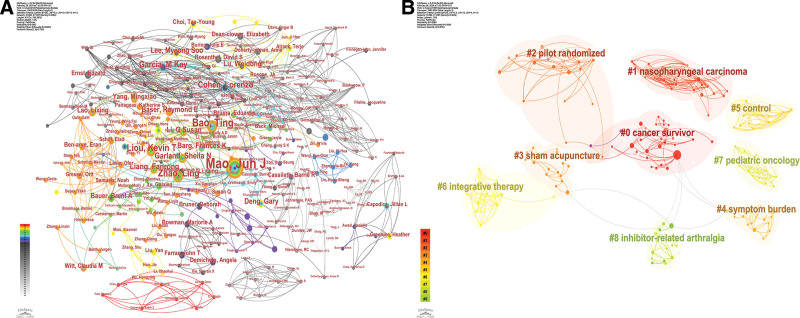
Author network analysis view. (A) Author co-occurrence analysis. (B) Author cluster analysis.

3067 authors contributed to the publications. Table 4 demonstrates the top 8 authors in terms of the number of publications, each of which has published at least 7 papers. Mao, Jun J published 38 papers and is the author with the highest number of publications, closely followed by Bao, Ting with 15 papers. Figure 5A shows the analytical view of the author network constructed based on the number of publications of each author, and the size of the node represents the number of publications. Mao, Jun J, and Zhao, Ling ranked the top 2 scholars in terms of centrality, both with a centrality of 0.02. This indicates that the research results of these 2 scholars have been recognized by researchers in the industry, and they have a high impact.

**Table 4:** Top 8 authors in terms of publication volume.

Rank	Count	Centrality	Authors
1	38	0.02	Mao, Jun J^[11]^
2	15	0.01	Bao, Ting
3	11	0.02	Zhao, Ling
4	10	0.01	Liou, Kevin T
5	8	0	Cohen, Lorenzo
6	8	0	Garcia, M Kay^[14]^
7	7	0.01	Liang, Fanrong
8	7	0.01	Garland, Sheila N^[15]^

Figure 5B, on the other hand, illustrates the results of the cluster analysis among authors with high publication volume, which form 10 more significant collaborative clusters, with the larger connecting points in each cluster indicating more publications by scholars in that cluster. The cluster with the highest heat was Cancer Survivor, followed by Nasopharyngeal Carcinoma and Pilot Randomized. This suggests that acupuncture is mainly used as an alternative and adjunctive therapy for the subsequent rehabilitation of cancer survivors. Acupuncture is primarily involved in the treatment of nasopharyngeal cancer. Scholars have conducted relevant studies mainly through randomized controlled trials.

#### 3.4.2. Analysis of co-cited authors

**Figure 6.**
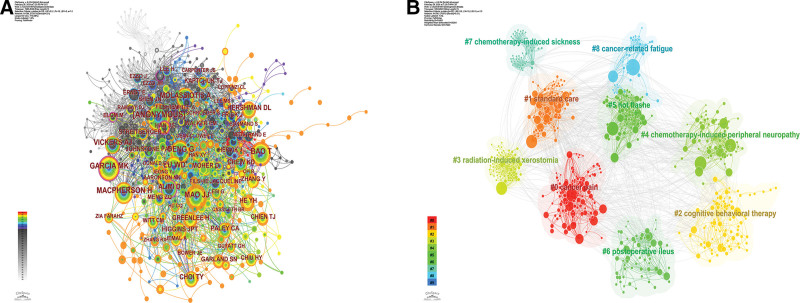
Network analysis view of cited authors. (A) Co-occurrence analysis view. (B) Clustering analysis view.

Table 5 shows the number of co-citations and centrality of the top 10 authors, and Figure 6A shows the co-occurrence analysis view of the co-cited authors, with the size of the nodes representing the number of citations. It can be found that Anonymous has the highest number of citations with 182 citations and a centrality of 0.07. The scholar with the 2nd highest number of citations is Mao JJ, with 130 citations and a centrality of 0.04, followed by Deng G, with 121 citations and a centrality of 0.09. Among the top 10 authors, the one with the highest centrality is Garcia MK, with 119 citations and 0.09 centrality. This indicates that although Garcia MK does not have the largest number of publications, his research is of high-quality and has a greater impact. Figure 6B shows the results of the cluster analysis of these authors, and it can be seen that these authors have formed a more significant cooperative relationship with each other, and the stable cooperative relationship promotes the progress of related research on this topic.

**Table 5:** Top 10 scholars with citation times.

Rank	Count	Centrality	Cited authors
1	182	0.07	Anonymous
2	130	0.04	Mao JJ
3	121	0.09	Deng G
4	119	0.11	Garcia MK
5	118	0.09	Lu WD
6	113	0.07	Bao T
7	112	0.07	Macpherson H
8	108	0.07	Molassiotis A
9	93	0.08	Vickers AJ

### 3.5. Journals analysis

**Figure 7.**
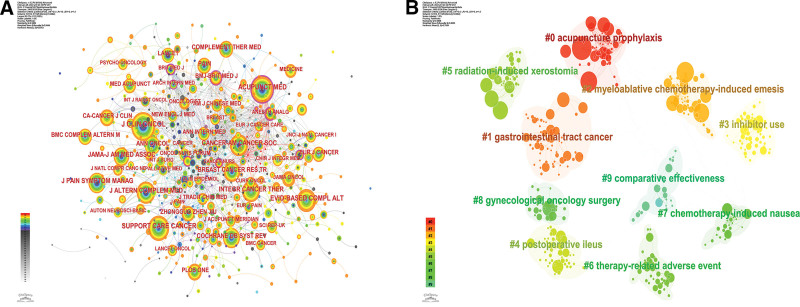
Network analysis view of cited journals. (A) Co-occurrence analysis view. (B) Cluster analysis view.

A total of 190 journals published publications on related topics. Table 6 demonstrates the top 10 most cited journals with centripetal and journal division, impact factor. Figure 7 demonstrates the cited journals, where the top 3 cited journals are J Clin Oncol, Acupunct Med, and Support Care Cancer, with 399, 306, and 287 citations, respectively. Combining the impact factor and partition, the latest impact factor of J CLIN ONCOL is 45.3 points and the partition is Q1, the latest impact factor of Acupunct Med is 2.5 points and the partition is Q3, and the latest impact factor of Support Care Cancer is 3.1 and the partition is Q1. 10 journals include the top journals JAMA-J Am Med Assoc, it can be seen that the research related to acupuncture for cancer treatment has high-quality research results published with authority. The results of cluster analysis showed that the cluster with the highest heat was acupuncture prophylaxis, followed by gastrointestinal tract cancer and myeloablative chemotherapy-induced emesis. This suggests that acupuncture in ameliorating cancer primarily plays a role in preventing gastrointestinal reactions as well as chemotherapy-induced adverse effects.

**Table 6:** Top 10 journals ranked in citation times.

Rank	Count	Centrality	Cited Journals	Division/IF
1	399	0.02	J Clin Oncol	Q1/45.3
2	306	0.22	Acupunct Med	Q3/2.5
3	287	0	Support Care Cancer	Q1/3.1
4	252	0.01	Evid-Based Compl Alt	/
5	236	0	J Altern Complem Med	Q3/2.6
6	229	0.02	Integr Cancer Ther	Q2/2.9
7	224	0.04	Cancer-Am Cancer Soc	Q1/6.2
8	206	0.04	J Pain Symptom Manag	Q1/4.7
9	200	0.04	JAMA-J Am Med Assoc	Q1/120.7
10	182	0.03	Cochrane Db Syst Rev	Q1/8.4

### 3.6. Analysis of references

**Figure 8.**
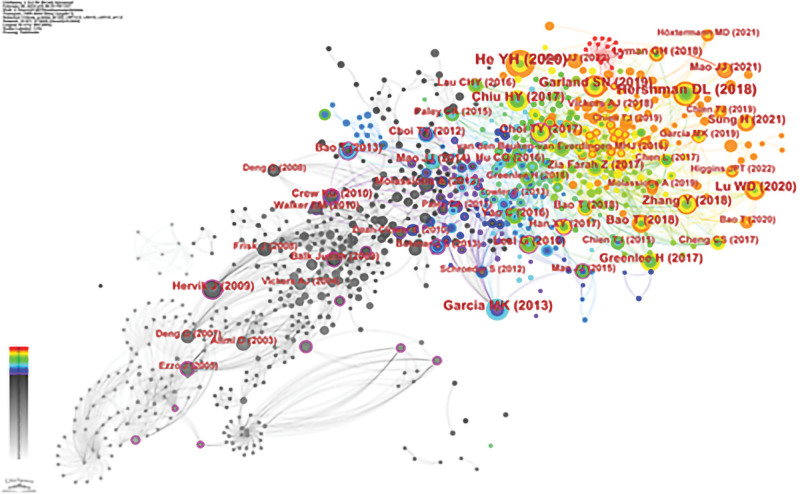
View of co-occurrence analysis of references.

Table 7 demonstrates the most co-cited literature.^[14–23]^ The network map of co-cited literature presented in Figure 8 is based on the references of 592 papers. There are a total of 911 co-citations in the 592 papers related to acupuncture for cancer improvement in the last 20 years. One of the most cited papers was published by He YH in JAMA Oncol, “Clinical Evidence for Association of Acupuncture and Acupressure With Improved Cancer Pain: A Systematic Review and Meta-Analysis.” This study provides evidence-based medical evidence that acupuncture combined with acupressure improves cancer-related pain. The most central piece of literature is “Acupuncture for Managing Cancer-Related Insomnia: A Systematic Review of Randomized Clinical Trials” by Choi TY published in INTEGR CANCER THER. Clinical Trials,” this study also provides evidence-based medical evidence for acupuncture for cancer-induced insomnia. It can be seen that the high-quality systematic review and meta-analysis were recognized by scholars and provided evidence support for related studies on this topic.

**Table 7:** The most cited references.

Rank	Count	Centrality	Cited reference
1	51	0.02	He YH, 2020, JAMA Oncol, V6, P271, DOI 10.1001/jamaoncol.2019.5233
2	41	0.06	Hershman DL, 2018, JAMA-J Am Med Assoc, V320, P167, DOI 10.1001/jama.2018.8907
3	31	0.05	Garcia MK, 2013, J Clin Oncol, V31, P952, DOI 10.1200/JCO.2012.43.5818
4	30	0.09	Garland SN, 2019, JNCI-J Natl Cancer I, V111, P1323, DOI 10.1093/jnci/djz050
5	29	0.07	Chiu HY, 2017, Eur J Cancer Care, V26, P0, DOI 10.1111/ecc.12457
6	26	0.03	Lu WD, 2020, Oncologist, V25, P310, DOI 10.1634/theoncologist.2019-0489
7	25	0.01	Zhang Y, 2018, Support Care Cancer, V26, P415, DOI 10.1007/s00520-017-3955-6
8	21	0.06	Greenlee H, 2017, CA-Cancer J Clin, V67, P195, DOI 10.3322/caac.21397
9	21	0.04	Bao T, 2018, Eur J Cancer, V101, P12, DOI 10.1016/j.ejca.2018.06.008
10	20	0.12	Choi TY, 2017, Integr Cancer Ther, V16, P135, DOI 10.1177/1534735416664172

### 3.7. Keyword analysis

#### 3.7.1. Keywords co-occurrence analysis

**Figure 9.**
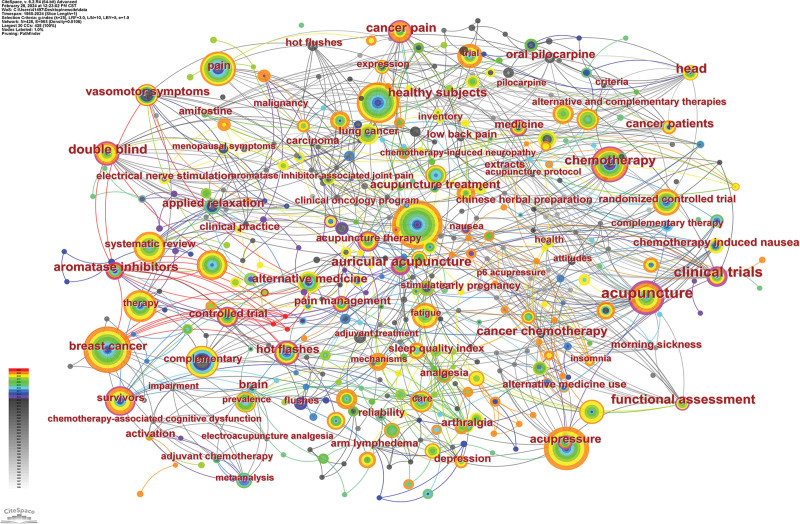
Keyword co-occurrence analysis view.

Table 8 demonstrates the keywords with the top 10 occurrences, none of the 10 keywords occurring no <56 times. The most frequently occurring keyword is breast cancer, which has a centrality of 0.07. The second most frequent keyword was management, which has a centrality of 0.03. The third most frequent keyword is quality of life, which has a centrality change of 0.01. The highest centrality of the 10 keywords is acupuncture. The focus of these keywords is centered on female patients and breast cancer, as well as alleviating complications during cancer treatment and improving patients’ quality of life through acupuncture. Figure 9 illustrates the co-occurrence analysis view of the keywords, where a larger area of the node indicates a higher number of occurrences.

**Table 8:** The keywords with the highest frequency of occurrence.

Rank	Count	Centrality	Keywords
1	145	0.07	Breast cancer
2	129	0.03	Management
3	115	0.01	Quality of life
4	113	0.02	Electroacupuncture
5	83	0.05	Pain
6	70	0.21	Acupuncture
7	67	0.01	Women
8	57	0.15	Chemotherapy
9	57	0.06	Complementary
10	56	0.05	Therapy

#### 3.7.2. Keywords clustering analysis

**Figure 10.**
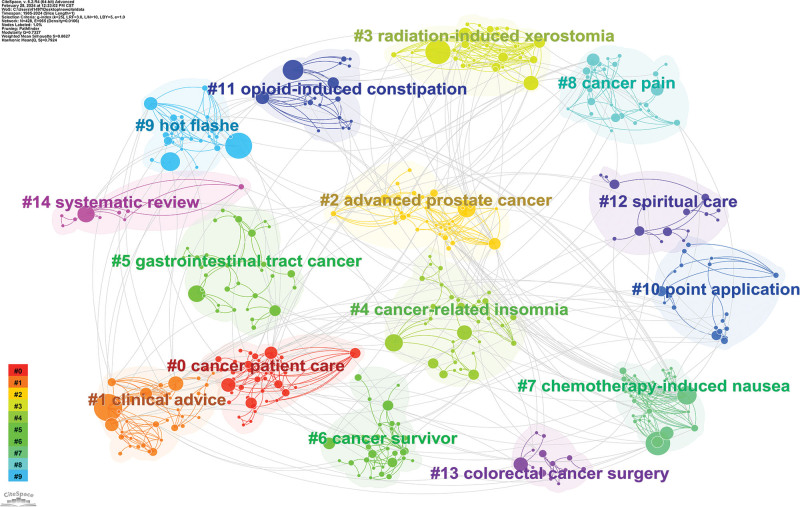
Keyword cluster analysis view.

Figure 10 shows the cluster analysis of the keywords which can be categorized into 15 significant clusters. The most popular cluster is cancer patient care, followed by clinical advice and advanced prostate cancer.

#### 3.7.3. Keywords timeline analysis

**Figure 11.**
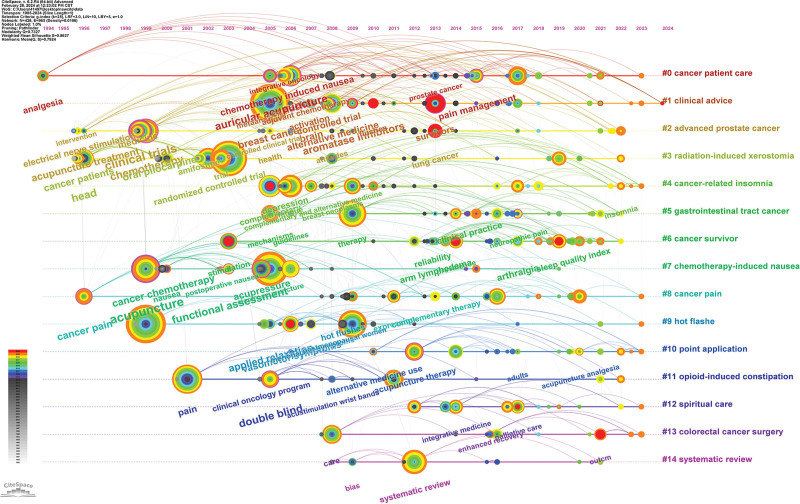
Keywords timeline analysis view.

Figure 11 shows the timeline visualization analysis of the keywords, reacting to the evolution of the keywords. It can be seen that scholars initially focused on the topic of cancer patient care, and the hotness of this keyword lasted from 1994 to today. The latest research hotspots focus on spiritual care, Colorectal Cancer Surgery, and systematic review.

## 4. Discussion

Based on the search results from the WOSCC, we analyzed the current and future trends in the use of acupuncture as an adjuvant therapeutic measure among cancers.

The analysis of countries|/regions shows that China and the United States have published the most literature in this area. There is a cooperative relationship between several countries, forming more significant clusters. China, South Korea, and England are at the center of their respective clusters.

The analysis of institutions showed that Memorial Sloan Kettering Cancer Center was the institution with the highest number of publications, and China Academy of Chinese Medical Sciences ranked 1st in centrality. The cluster analysis of the institutions formed stable clusters, each with a distinct theme, with the hottest theme being cancer-related fatigue.

The results of the authors analysis showed that Mao, Jun J, and Bao, Ting were the 2 authors with the highest number of publications. The 2 scholars with the highest centrality are Mao, Jun J, and Zhao, Ling. The results of the authors’ clustering analysis show that the current scholars are mainly focusing on studies related to cancer survivors, nasopharyngeal carcinoma, and pilot randomized. Anonymous is the most cited author and Garcia MK is the most centrally cited author.

The analysis of cited journals shows that J Clin Oncol (Q1/45.3) is the most cited journal. The top 10 cited journals included top journals JAMA-J Am Med Assoc. this suggests that the research on this topic has achieved high-quality results and is authoritative. The results of cluster analysis suggest that acupuncture prophylaxis, gastrointestinal tract cancer, and myeloablative chemotherapy-induced emesis are the research topics of greater interest to scholars.

Analysis of the references revealed 592 documents with 911 co-references and the highest number of citations at 51, published in JAMA Oncol. This publication focuses on the results of a systematic evaluation and meta-analysis of acupuncture combined with acupressure for the amelioration of pain associated with cancer, which provides evidence-based support for research on this topic.

The results of the keyword analysis demonstrated the research directions that scholars focus on in this field. breast cancer is the keyword with the highest frequency of occurrence. Among the top 10 keywords with the highest frequency of occurrence, we found both quality of life, Pain, and therapy. these keywords demonstrated the current major focus of acupuncture to improve cancer. Cluster analysis revealed that the keywords could be clustered into 15 clusters, with cancer patient care being the most popular cluster. Timeline analysis showed spiritual care, Colorectal Cancer Surgery, and systematic review as the most recent themes.

## 5. Limitation

Although we tried to maintain the rigor of this study as much as possible, certain limitations remain. We only included articles from the WOS core library and therefore did not guarantee the inclusion of all studies on this topic. Due to the extensive nature of the search measurement strategy, we manually screened the retrieved literature. Therefore, the literature included in this study is sufficiently accurate but may not be comprehensive.

## 6. Conclusion

This study found that research under this theme has focused on the application of acupuncture therapy to improve the quality of life of cancer patients, with positive findings in the improvement of cancer-induced pain and insomnia. In addition. We found that spiritual care for cancer patients, postoperative adverse effects of colorectal cancer, and systematic review became new research hotspots, which suggests future research trends. In conclusion, acupuncture has a positive role and prospects as a complementary and alternative therapy in improving cancer-related symptoms.

## Acknowledgments

We thank those who participated in and supported this study.

## Author contributions

**Conceptualization:** Rui Shang, Yunchen Song.

**Data curation:** Fengyang Wang, Xi Nan.

**Formal analysis:** Xi Nan.

**Investigation:** Ting Pan, Fengyang Wang.

**Methodology:** Rui Shang.

**Supervision:** Hui Jin, Yunchen Song.

**Validation:** Ting Pan, Hui Jin.

**Visualization:** Hui Jin.

**Writing – original draft:** Rui Shang.

**Writing – review & editing:** Ting Pan, Yunchen Song.
